# Preliminary Characterization of MEDLE-2, a Protein Potentially Involved in the Invasion of *Cryptosporidium parvum*

**DOI:** 10.3389/fmicb.2017.01647

**Published:** 2017-08-31

**Authors:** Baoling Li, Haizhen Wu, Na Li, Jiayuan Su, Ruilian Jia, Jianlin Jiang, Yaoyu Feng, Lihua Xiao

**Affiliations:** ^1^State Key Laboratory of Bioreactor Engineering, School of Resources and Environmental Engineering, East China University of Science and Technology Shanghai, China; ^2^School of Biotechnology, East China University of Science and Technology Shanghai, China; ^3^College of Veterinary Medicine, South China Agricultural University Guangzhou, China; ^4^Emory Vaccine Center, Yerkes National Primate Research Center, Emory University, Atlanta GA, United States; ^5^Division of Foodborne, Waterborne, and Environmental Diseases, National Center for Emerging and Zoonotic Infectious Diseases, Centers for Disease Control and Prevention, Atlanta GA, United States

**Keywords:** *Cryptosporidium parvum*, MEDLE family, invasion, expression, antigen

## Abstract

*Cryptosporidium* spp. are important causes of diarrhea in humans, ruminants, and other mammals. Comparative genomic analysis indicated that genetically related and host-adapted *Cryptosporidium* species have different numbers of subtelomeric genes encoding the *Cryptosporidium*-specific MEDLE family of secreted proteins, which could contribute to differences in host specificity. In this study, a *Cryptosporidium parvum*-specific member of the protein family MEDLE-2 encoded by cgd5_4590 was cloned and expressed in *Escherichia coli*. Immunofluorescent staining with antibodies generated from the recombinant protein showed the expression of the protein in sporozoites and development stages. *In vitro* neutralization assay with the antibodies partially blocked the invasion of sporozoites. These results support the potential involvement of MEDLE-2 in the invasion of host cells.

## Introduction

*Cryptosporidium* spp. are important pathogens inhabiting the brush borders of the gastrointestinal epithelium of various vertebrates, causing enterocolitis, diarrhea, and vomiting (Checkley et al., 2015). Of the near 100 recognized *Cryptosporidium* species and genotypes, most can infect only a few closely related hosts (Ryan et al., 2014). For example, *C. andersoni* and *C. bovis* infect mainly cattle, *C. hominis* and *C. viatorum* infect mostly humans, and *C. canis* and *C. felis* infect dogs and cats, respectively. Some of the species, such as *C. parvum, C. meleagridis* and *C. ubiquitum* have a broad host range, thus are considered important zoonotic parasites (Ryan et al., 2014). Among them, *C. parvum* has attracted public health attention because of its high pathogenicity in ruminants and high infectivity to humans.

The biologic determinants for host specificity in *Cryptosporidium* spp. are not clear. This is largely caused by our poor knowledge of major components involved in the invasion of host cells by sporozoites (Liu et al., 2016). Efforts have been made in the identification of proteins that are potentially involved in host cell invasion by screening cDNA library using immune sera. A few of these proteins identified are considered potentially involved in *Cryptosporidium* invasion, such as gp900, gp40/15 (gp60), Cpa135, Cp2, P23, and TRAP-C1 (Bouzid et al., 2013). Most of these proteins are *O*-glycosylated and many of them are mucins. As the invasion process is considered to be conserved among apicomplexan parasites and these proteins are rarely seen in non-*Cryptosporidium* apicomplexans, other proteins could also be involved in invasion and host specificity in *Cryptosporidium* spp.

Comparative genomic analysis of *C. parvum* and *C. hominis*, which differ from each at the nucleotide level by merely ∼3% but have very different host range, indicates that these two species have different copies of subtelomeric genes encoding two major protein families (Guo et al., 2015). One of the protein families involved is the *Cryptosporidium*-specific MEDLE family of secreted proteins at the 3′ end of chromosomes 5 and 6 (Guo et al., 2015). *C. parvum* has six paralogous genes encoding MEDLE proteins, compared with one in *C. hominis*. As host-adapted *C. parvum* IIa (infecting mostly cattle) and IId (infecting mostly sheep and goats) subtype families also differ in the number of MEDLE genes (Feng et al., 2017), it was suggested that MEDLE proteins could contribute to differences in host specificity among *Cryptosporidium* spp.

In this study, we conducted a preliminary biologic study of the *C. parvum*-specific MEDLE-2 encoded by the cgd5_4590 gene. The aim of this study was to express the protein in *Escherichia coli*, assess its expression on sporozoites and developmental stages in culture, and evaluate its potential role in host cell invasion.

## Materials and Methods

### *Cryptosporidium parvum* Isolate, Host Cells, *E. coli* Strains and Plasmid Vectors

*Cryptosporidium parvum* oocysts (IOWA strain) were purchased from Waterborne, Inc. (New Orleans, LA, United States) and stored in antibiotics at 4°C for less than 2 months prior to use. Before experiments, oocysts were treated with 0.5% sodium hypochlorite on ice for 10 min and washed 3 times with sterile PBS. Human ileocecal adenocarcinoma HCT-8 cells were obtained from Chinese Academy of Sciences Shanghai Branch. Cells were cultured in maintenance medium at 37°C in a humidified atmosphere containing 5% CO_2_. *E. coli* strains DH5α and BL21 (DE3) (Tiangen, Beijing, China) were used for plasmid amplification and expression, respectively. The pET28a vector was obtained from Novagen, Inc. (Madison, WI, United States). All restriction enzymes were purchased from Thermo Fisher Scientific, Inc. (Waltham, MA, United States).

### Construction of Recombinant Plasmid

The cgd5_4590 gene (XM_625307) was amplified by PCR from *C. parvum* DNA and cloned into the pET28a vector as an *Nco*I*-Xho*I insertion using the following primers: forward 5′-AAATCCATGGAAAATGTAACCGATAATT-3′ (including an *Nco*I restriction site) and reverse 5′-AAATCTCGAGTTCCAAATCATGAAGAATATC-3′ (including an *Xho*I restriction site). The 50 μl-PCR reaction contained 1 μl of DNA, 0.25 mM primers, 3 mM MgCl2, 200 μM deoxynucleotide triphosphates, 1× GeneAmp PCR buffer (Applied Biosystems, Foster City, CA, United States), and 1.5 U of Taq polymerase (Promega, Madison, WI, United States). The amplification was performed on a GeneAmp 9700 (Applied Biosystems), consisting of an initial denaturation at 94°C for 5 min; 35 cycles at 94°C for 45 s, 52°C for 45 s, and 72°C for 1 min; and a final extension at 72°C for 7 min. The template DNA was extracted from *C. parvum* oocysts by using the Qiagen DNeasy Blood & Tissue Kit (Qiagen, Hilden, Germany). The target gene is located in the 3′ subtelomeric region of chromosomes 5, encoding the MEDLE-2 protein. The molecular weight of the expected protein is 21.0 kDa, with no predicted signal peptide, transmembrane domain, or glycosylphosphatidylinositol anchor. It has 16 predicted *O*-linked glycosylation sites, mostly concentrated between amino acids 48–69 and 104–117.

The PCR product was purified using the SanPrep Column PCR Product Purification Kit (Sangon Biotech, Shanghai, China), digested with *Nco*I and *Xho*I and ligated into the expression vector pET28a. The recombinant vector cgd5_4590-pET28a was introduced into competent *E. coli* DH5α and transformed cells were selected on LB ager with 50 μg/ml of kanamycin. Positive colonies were randomly picked and identified by PCR. The PCR products from these colonies were sequenced to verify their identity and sequence accuracy.

### Expression of Recombinant MEDLE-2 Protein

The recombinant plasmid was extracted from *E. coli DH5*α using the EZ-10 Spin Column Plasmid DNA Minipreps Kit (Sangon Biotech) and introduced into *E. coli* BL21 (DE3) to express the target protein. The *E. coli* BL21 (DE3) cells harboring the recombinant plasmid were cultured at 37°C until OD_600_ reaching 0.6–1.0, by which time 0.1 mM isopropyl b-D-1-thiogalactopyranoside (IPTG) was added to induce protein expression. The induction was conducted at 18, 25, and 37°C for 4 h to select the optimal expression condition. The expression level and solubility of the target protein were compared among induction temperatures by SDS–PAGE and Western blot analyses of the bacterial cells harvested at OD_600_ = 1.0.

### SDS–PAGE and Western Blot Analyses

The bacterial cells were harvested by centrifugation and lysed by boiling in 5× protein loading buffer for 5 min. Proteins in 20 μL lysate were separated by 10% SDS–PAGE and stained with Coomassie Brilliant Blue (Bio-Rad, Hercules, CA, United States).

For Western blot analysis of the recombinant MEDLE-2, proteins resolved by 10% SDS–PAGE were transferred onto a polyvinylidene fluoride (PVDF) membrane using a semi-dry electro-blotting apparatus (Bio-Rad). The transfer was carried out at 400 mA for 1 h. After blocking with TBST containing 5% non-fat milk at room temperature for 2 h, the membrane was incubated overnight with 1:1,000 anti-His tag antibodies (Cell Signaling Technology, Danvers, MA, United States). Afterwards, the PVDF membrane was washed three times with TBST and incubated at room temperature with 1:5,000 goat anti-mouse IgG-A (HRP) antibodies (Yeasen, Shanghai, China) for 2 h. The membrane was finally washed three times with TBST and reactive protein bands in the membrane were detected using the DAB kit (Tiangen Biotech).

For Western blot analysis of native proteins of *C. parvum* sporozoites, 1 × 10^8^ oocysts were in PBS buffer and Protease Inhibitor Cocktail Set III (Calbiochem, La Jolla, CA, United States) were sonicated on ice for 30 min. The lysis was collected by centrifugation at 6,000 *g* for 30 min. The soluble native proteins and recombinant protein were mixed with 5× protein loading buffer, heated at 100°C for 5 min, and subjected to Western blot analysis using pre-immune serum, immune serum, and purified polyclonal antibodies to recombinant MEDLE-2.

### Purification of MEDEL-2 and Preparation of Polyclonal Antibodies

For the purification of MEDEL-2, *E. coli* cells were inoculated into 2-L culture with induction of protein expression by 1 mM IPTG at 25°C for 4 h. The cells were harvested by centrifugation, re-suspended in PBS buffer, and lysed by sonication on ice. The soluble fraction of the *E. coli* lysate was collected by centrifugation at 6,000 *g* for 20 min and filtered through a 0.45 μm cellulose acetate membrane filter (Millipore, Billerica, MA, United States). The filtrate was loaded onto Ni-NTA beads (Novagen, Madison, WI, United States) at 4°C and 100 rpm for 2 h. The beads were washed with 8 volumes of washing buffer containing 20 mM imidazole and MEDEL-2 was eluted off the beads with buffer containing 100, 200, or 250 mM imidazole. The final product was analyzed by SDS–PAGE. The purified MEDLE-2 protein was used in the production of polyclonal antibodies in rabbits by GL Biochem Ltd. (Shanghai, China). For this, 350 μg of MEDLE-2 protein mixed with Freund’s complete adjuvant was used in the immunization of healthy New Zealand white rabbits on Days 1 and 15. Immunized animals were boosted six times with 150 μg of MEDLE-2 protein in the Freund’s incomplete adjuvant every 7 days. Seven days after the last immunization, sera were collected from the immunized animals and the polyclonal IgG antibodies were purified from the immune sera using protein A sepharose affinity chromatography.

### Localization of MEDLE-2 Expression on *C. parvum* Sporozoites and Developmental Stages

Sporozoites on slides and intracellular stages of *C. parvum* in HCT-8 cell cultures at 24 and 48 h were fixed at room temperature for 30 min with 4% paraformaldehyde. Afterwards, the slides were washed three times with PBS for 3 min. About 250 μL of DB Blocking Buffer (B100-40, Waterborne, Inc.) was added to the slides. After incubation at 37°C for 30 min and three washes with the buffer, the slides were incubated with affinity-purified anti-MEDLE-2 antibodies (0.3 μg/ml) for 1 h, washed three times with PBS, and incubated with 1:100 Alexa Fluor 488-conjugated AffiniPure Goat Anti-Rabbit lgG (Yeasen) for 1 h (Jakobi and Petry, 2006). After three washes with PBS, 4′, 6-diamidino-2-phenylindole (DAPI, Roche, Basel, Switzerland) was used to counterstain the nuclei of sporozoites and developmental stages. The slides were mounted with No-Fade Mounting Medium (Boster, Wuhan, China) and examined under a BX53 immunofluorescence microscope (Olympus, Tokyo, Japan).

### Examination of CGD5_4590 Gene Expression by QPCR

The expression of the cgd5_4590 gene in parasites developing in HCT-8 cells at 0–72 h was evaluated by qPCR (Mauzy et al., 2012). The expression of 18S rRNA gene was determined in parallel for data normalization (Cai et al., 2005). Each 20 μL-qPCR reaction contained 0.1 mM primers, 4 μL of 1:10 dilution of cDNA synthesized from 2 μg RNA using RevertAid First Strand cDNA Synthesis Kit (Thermo Fisher Scientific) and SsoFast EvaGreen Supermix (Bio-Rad). The qPCR was conducted on a Light Cycler 480 (Roche), with an initial denaturation at 95°C for 1 min and 45 cycles of 95°C for 30 s, 58°C for 30 s, and 68°C for 30 s. The amplification was followed by a melt curve analysis through 95°C for 1 min, ramping down to 57°C in 2 degree intervals, and ramping back to 95°C for 30 s. The primers used were 5′-AGGTAGGGGTGGAGGAGGTA-3′ and 5′-ATGGCTCATCAAATGGGTCT-3′ for the cgd5_4590 gene (amplicon size = 185 bp), and 5′-AGGTAGGGGTGGAGGAGGTA-3′ and 5′-ATGGCTCATCAAATGGGTCT-3′ for the 18S rRNA gene (amplicon size = 256 bp) (Wang et al., 2013; Guo et al., 2016). Threshold cycle (C_T_) values from qPCR were used in computing the relative levels of expression, in which data were first normalized by computing the ΔC_T_ values between cgd5_4590 mRNA (C_T__[sample]_) and 18S rRNA (C_T__[Cp18S]_) for all samples using the formula:

$$\Delta {\text{C}}_{\text{T}}={\text{C}}_{\text{T}\left[\text{sample}\right]}-{\text{C}}_{\text{T}\left[\text{Cp18S}\right]}$$

Next, ΔC_T_ values (relative levels of *C. parvum* 18S rRNA) in each experiment were further normalized by subtracting the ΔC_T0_ values (ΔC_T_ at 0 h point):

$$\Delta \Delta {\text{C}}_{\text{T}}=\Delta {\text{C}}_{\text{Tn}}-\Delta {\text{C}}_{\text{T0}}\;(\Delta {\text{C}}_{\text{Tn}}\;\text{where}\;n\;=\;0,\;2,\;6,\;12,\;24,\;36,\;48,\;72)$$

The relative expression of the cgd5_4590 gene was calculated using the 2^-ΔΔC^_T_ method (Livak and Schmittgen, 2001). Mean values from at least three independent experiments were obtained.

### *In Vitro* Neutralization of Sporozoite Invasion

A neutralization assay was used to assess the potential involvement of MEDLE-2 in host cell invasion by *C. parvum* sporozoites in a BSL-2 laboratory (Ludington and Ward, 2016). HCT-8 cells were grown in 12-well plates to 80–90% confluence in RPMI 1640 medium supplemented with 10% fetal bovine serum (FBS), 1 mM sodium pyruvate, 50 U/ml penicillin G, 50 U/ml streptomycin, and 0.25 mg/ml amphotericin B (pH 7.4). Oocysts were treated with 0.5% sodium hypochlorite on ice for 10 min and washed 3 times with cold sterile PBS (Gut and Nelson, 1999). For neutralization assays, 1 × 10^5^ oocysts were incubated with different dilutions of immune serum or pre-immune serum in infection medium in HCT8 cell culture at 37°C for 2 h. Based on results of preliminary evaluations, 1:50, 1:100, and 1:500 dilutions of sera were used in neutralization assays. After the 2-h incubation, the cultures were washed off free sporozoites and incubated for additional 48 h. Cy3-labeled polyclonal antibodies against *C. parvum* sporozoites (Waterborne, Inc.) were used to determine the infection rate of HCT-8 cells as described (Upton et al., 1995; Deng et al., 2002). Images taken under 200× were digitalized and the relative staining area was quantified by using ImageJ^1^. The mean parasite load in each culture was calculated based on images from 30 random fields, and compared between treatment groups using the Mann–Whitney *U*-test. The inhibition rate of sporozoite invasion was calculated based on differences in parasite load between MEDLE-2 antibody treated and control groups.

## Results

### Cloning of CGD5_4590 Gene and Expression of Recombinant MEDLE-2 Protein

**FIGURE 1:**
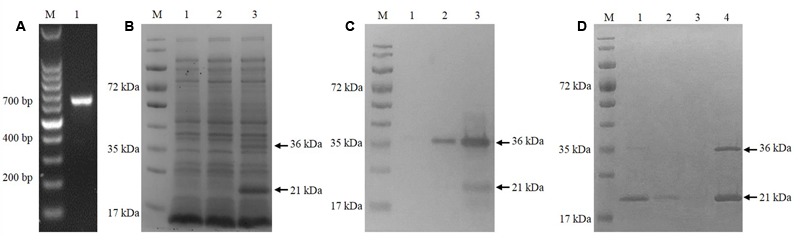
Expression of recombinant MEDLE-2 protein in *Escherichia coli*. **(A)** PCR amplification of the cgd5_4590 gene in *C. parvum*. Lane M: 100-bp molecular makers; Lanes 1: cgd5_4590 PCR product. **(B)** Expression of recombinant MEDLE-2 protein in *E. coli* BL21 (DE3) revealed by SDS–PAGE analysis. Lane M: protein size makers; Lane 1: lysate from bacterial cells transformed with pET28a vector control; Lane 2: lysate from bacterial cells transformed with pET28a-cgd5_4590 without IPTG induction; Lane 3: lysate from bacterial cells transformed with pET28a-cgd5_4590 with IPTG induction. **(C)** Expression of recombinant MEDLE-2 protein in *E. coli* BL21 (DE3) revealed by Western blot analysis using Anti-His tag. Lane M: protein size makers; Lane 1: lysate from bacterial cells transformed with pET28a vector control; Lane 2: lysate from bacterial cells transformed with pET28a-cgd5_4590 without IPTG induction; Lane 3: lysate from bacterial cells transformed with pET28a-cgd5_4590 with IPTG induction. **(D)** Purity of recombinant MEDLE-2 revealed by SDS–PAGE analysis. Lane M: protein size makers; Lane 1: the first volume of washing solution with 20 mM imidazole from the purification column; Lane 2: the fifth volume of washing solution with 20 mM imidazole from the purification column; Lane 3: the eighth volume of washing solution with 20 mM imidazole from the purification column; Lane 4: target protein eluted from the purification column using 250 mM imidazole.

The cgd5_4590 gene was amplified by PCR using specific primers (**Figure 1A**) and successfully cloned into the pET28a vector using the T4 DNA ligase. After verification of the identity and sequence by DNA sequencing, the recombinant plasmid was introduced into *E. coli* BL21 (DE3) for MEDLE-2 protein expression. The profile of total proteins from the negative control culture, transformed culture with no induction of MEDLE-2 expression, and transformed culture with induction was analyzed by 12% SDS–PAGE, which showed the presence of two unique protein bands of 21 and 36 kDa size in total proteins from transformed cultures (**Figure 1B**). This was confirmed using Western blot analysis using anti-His tag antibodies (**Figure 1C**).

**FIGURE 2:**
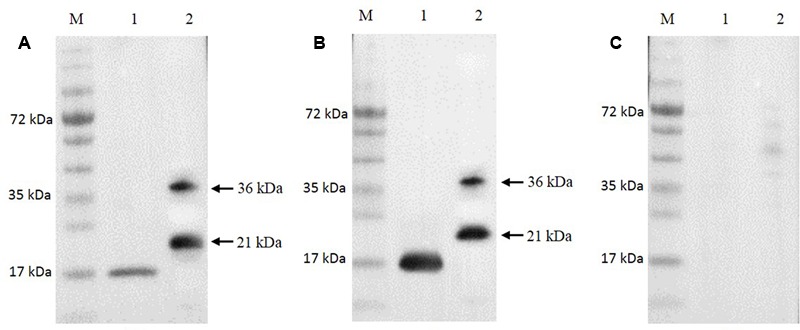
Expression of native MEDLE-2 protein in *Cryptosporidium parvum* sporozoites. Polyclonal IgG antibodies **(A)**, immune serum **(B)** and pre-immune serum **(C)** were used in Western blot analysis of total proteins extracted from *C. parvum* oocysts and recombinant proteins. Lane M: protein size makers; Lane 1: native proteins from *C. parvum*; Lane 2: recombinant MEDLE-2.

The Ni-NTA purification method was used to obtain highly pure recombinant MEDLE-2 protein (**Figure 1D**). The target protein was eluted by using 250 mM imidazole. The MEDLE-2 identity of the purified proteins was established by using the time-of-flight mass spectrometry; both 21 and 36 kDa proteins yielded peptide sequences compatible to the MEDLE-2 sequence (Accession No. XM_625307; data not shown). In western blot analysis, the recombinant MEDLE-2 protein was expectedly recognized by immune serum or purified antibodies from rabbits immunized with MEDLE-2 (**Figures 2A,B**), but not the pre-immune serum (**Figure 2C**). The immune serum and purified antibodies also recognized a native protein of approximately 17 kDa from sporozoites.

### Localization of MEDLE-2 Protein

**FIGURE 3:**
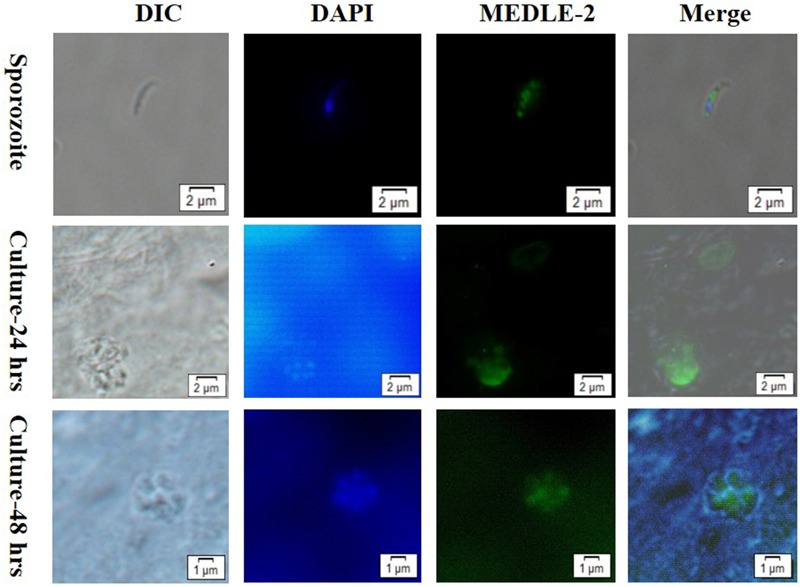
Localization of MEDLE-2 protein expression on *Cryptosporidium parvum* sporozoites **(Top)** and developmental stages of *C. parvum* in HCT-8 cell cultures at 24 and 48 h (**Middle**, **Bottom**, respectively). Images were taken by differential interference contrast microscopy (DIC), fluorescence microscopy using nuclear stain 4, 6-diamidino-2-phenylindole (DAPI), fluorescence microscopy using polyclonal MEDLE-2 antibodies (MEDLE-2), and superimposition of the three (Merge).

The expression of MEDLE-2 protein on sporozoites and developmental stages of *C. parvum* in HCT-8 cell cultures at 24 and 48 h were examined by immunofluorescence. The staining of sporozoites with anti-MEDLE-2 polyclonal antibodies showed a diffused expression of MEDLE-2 within sporozoites, including the anterior end. The highest MEDLE-2 expression was in the area just above the nucleus, which was located at the 1/3 posterior end of sporozoites by DAPI staining (**Figure 3**, Top). The immunofluorescence staining of developmental stages in 24 and 48 h HCT-8 cultures showed the expression of MEDLE-2 proteins on both the parasitophorus vacuoles of meronts and merozoites within them (**Figure 3**, Middle, Bottom).

### Expression of MEDLE-2 Gene in *C. parvum* Culture

**FIGURE 4:**
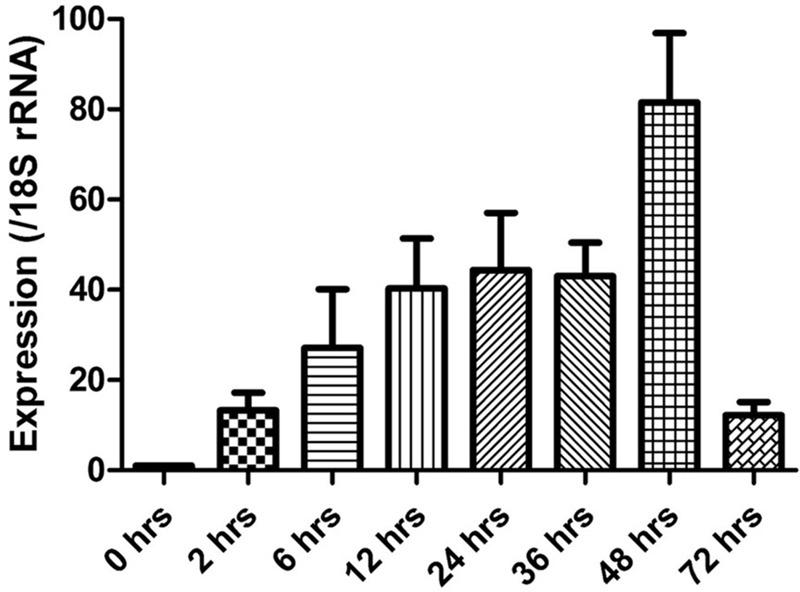
Relative expression of the cgd5_4590 gene in HCT-8 cell culture of *Cryptosporidium parvum* after the normalization of Ct values of qPCR with data from the 18S rRNA gene of *C. parvum.* Data shown are mean ± SD from three replicate assays.

The expression of the cgd5_4590 gene in *C. parvum* culture was assessed by qPCR, after data normalization using Ct values from qPCR analysis of the 18S rRNA gene of *C. parvum*. Newly released sporozoites had minimal expression of the cgd5_4590 gene. After the invasion, the expression of the cgd5_4590 gene increased gradually, with the highest expression level detected at 48 h of culture. A very low level of cgd5_4590 gene expression was seen at 72 h of culture (**Figure 4**).

### Neutralization of Sporozoite Invasion by MEDLE-2 Antibodies

**FIGURE 5:**
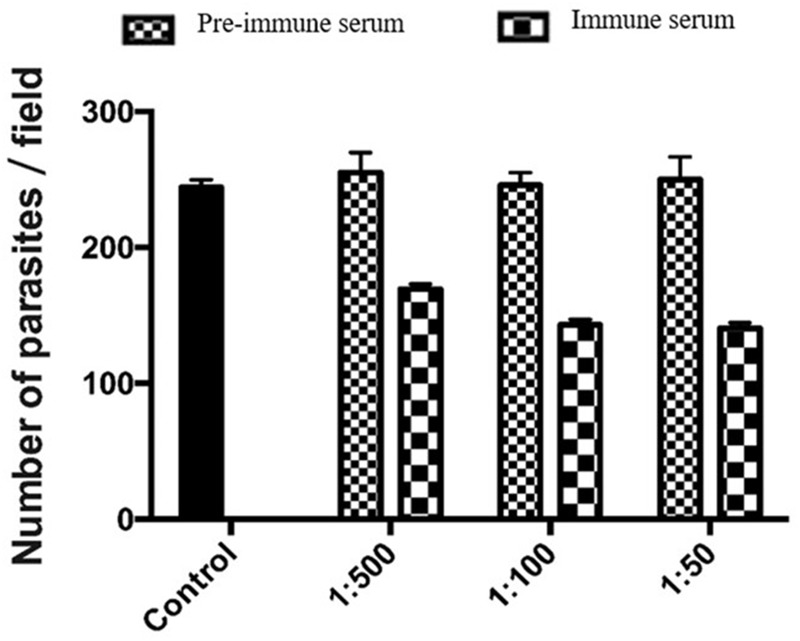
Inhibition of *Cryptosporidium parvum* invasion of HCT-8 cells by polyclonal MEDLE-2 antibodies. Data presented are mean ± SD parasites per 200 X field based on numbers from 30 randomly selected microscopy fields of *C. parvum* cultures. Control, *C. parvum* culture maintained in growth medium alone; 1:500, *C. parvum* culture with the addition of pre-immune or immune serum 1:500 dilution; 1:100, *C. parvum* culture with the addition of pre-immune or immune serum 1:100 dilution; 1:50, *C. parvum* culture with the addition of pre-immune or immune serum 1:50 dilution.

We used an invasion neutralization assay to assess the role of MEDLE-2 protein in the infection of intestinal epithelial cells by *C. parvum*. When HCT-8 cells were infected with sporozoites from 1 × 10^5^ oocysts, comparable parasite loads were obtained 48 h after the invasion between the control cultures and cultures with 1:50, 1:100, and 1:500 dilutions of the pre-immune serum. The mean parasite load in HCT-8 culture without the addition of any serum was 233.6 ± 35.6 per 200 X field. The addition of 1:500 dilution of the immune serum reduced the parasite load in HCT-8 cultures by 33.6% (255.1 ± 57.2 and 169.3 ± 29.2 per 200 X field for the pre-immune serum and immune serum groups, respectively; *P* = 0.000). At the 1:100 dilution, the inhibition rate of sporozoite invasion by MEDLE-2 immune serum was 41.8% (246.0 ± 46.8 and143.2 ± 38.7 per 200 X field for the pre-immune serum and immune serum groups, respectively; *P* = 0.000). At the 1:50 dilution, the inhibition rate of sporozoite invasion by MEDLE-2 immune serum was 43.8% (250.1 ± 46.8 and 140.6 ± 35.8 per 200 X field for the pre-immune serum and immune serum groups, respectively; *P* = 0.000) (**Figure 5**).

## Discussion

Results of the study support the previous suggestion on the potential involvement of MEDLE proteins in the invasion of *Cryptosporidium* spp. This is based on the expression of the MEDLE-2 protein in *C. parvum* sporozoites and merozoites, the expression of the cgd5-4590 gene in multiple developmental stages of the parasite, and the significant reduction of parasite load by polyclonal MEDLE-2 antibodies in *in vitro* cultures. As in studies of other invasion-related proteins of *Cryptosporidium* spp. (Barnes et al., 1998; Cevallos et al., 2000; O’Connor et al., 2009), only a partial blockage of the invasion of sporozoites was produced by anti-MEDLE-2 antibodies. This is expected, as apicomplexans have been long known to use multiple strategies to invade host cells (Sibley, 2004). Like some other *Cryptosporidium* proteins involved in invasion (Strong et al., 2000), native MEDLE-2 appears to be *O*-glycosylated, although as not heavily. The neutralization ability of MEDLE-2 antibodies could be enhanced if the recombinant protein is expressed in a eukaryotic system that would produce proteins with similar *O*-linked glycosylation.

The function of MEDLE proteins is not clear. The MEDLE family of secreted proteins were initially identified through whole genome sequencing of *C. parvum* (Abrahamsen et al., 2004). They have significant sequence similarity to each other, and most of them end with the amino acid sequence MEDLE. As they have no conserved protein domains and no apparent counterparts in other apicomplexan parasites, MEDLE proteins are considered *Cryptosporidium*-specific (Abrahamsen et al., 2004). The biologic importance of MEDLE proteins is reflected by their numbers; *C. parvum* was identified to have six genes encoding the MEDLE proteins but only one copy of genes encoding most other proteins. The potential involvement of MEDLE proteins in host specificity and invasion of *Cryptosporidium* spp. was suggested by comparative genomic analysis of two genetically related species *C. parvum* and *C. hominis*, as the species with a broad host range, *C. parvum*, has six MEDLE genes, compared with one MEDLE gene in *C. hominis*, which mainly infects humans and non-human primates (Guo et al., 2015; Liu et al., 2016).

In this preliminary study, we have focused on the characterization of MEDLE-2. This protein was chosen as the first MEDEL protein to study largely because of its divergent sequence compared with other MEDLE proteins. While other MEDLE proteins share 55–75% amino acid sequence similarities, MEDLE-2 has <30% sequence identity to them. This is especially the case in the second half of the protein, which has minimal sequence identity to other MEDLE proteins and ends with LHDLE instead of MEDLE. This makes gene cloning and protein characterization of MEDLE-2 simpler.

The subcellular location of MEDLE-2 is not clear. Although MEDLE proteins are all called secreted proteins, it appears that only MEDEL-5 and MEDLE-6 encoded by subtelomeric genes in chromosome 6 have the predicted signal peptide. The native protein of MEDLE-2 is smaller than expected (17 kDa versus 21 kDa), indicating that it could be processed after its translation. Thus, different MEDLE proteins may have different subcellular locations and function differently. Further immunochemistry and biologic studies are needed to understand the function of MEDLE-2 and other MEDLE proteins. Studies are also needed to determine whether the function of these antigens is dependent on the presence of *O*-linked alpha-N-acetylgalactosamines.

## Author Contributions

BL, JS, RJ, JJ, and NL conducted the experiments. BL, HW, YF, and LX analyzed the data and wrote the paper.

## Conflict of Interest Statement

The authors declare that the research was conducted in the absence of any commercial or financial relationships that could be construed as a potential conflict of interest.

## Footnotes

1 https://imagej.nih.gov/ij/
